# Sustainable remediation of heavy metal contaminated soil through phytostabilization with the *in-situ* immobilization by mercapto-based palygorskite

**DOI:** 10.3389/fpls.2025.1659418

**Published:** 2025-10-24

**Authors:** Hongjie Kuang, Bin Wu, Fengge Peng, Yahui Yu, Xiaohui Ma, Yuxian Shangguan

**Affiliations:** ^1^ State Key Laboratory of Geohazard Prevention and Geoenvironment Protection, College of Ecology and Environment, Chengdu University of Technology, Chengdu, China; ^2^ Institute of Agricultural Resources and Environment, Sichuan Academy of Agricultural Sciences, Chengdu, China

**Keywords:** mercapto-based palygorskite, Cd and Pb contamination, heavy metal immobilization, *Chrysopogon zizanioides*, microbial diversity

## Abstract

Phytostabilization has been widely applied to remediate mining soils contaminated with heavy metals, but the high soil toxicity often restricts plant growth and remediation efficiency. In this experiment, we investigated the effect of mercapto-based palygorskite (MPAL, applied at 2% and 4% w/w) on cadmium (Cd) and lead (Pb) phytostabilization by *Chrysopogon zizanioides* in soils contaminated with Cd and Pb. The results showed that soil pH did not vary under the application of MPAL but decreased during the cultivation of *C. zizanioides*. Compared with planting *C. zizanioides* alone, the application of MPAL significantly promoted the growth of *C. zizanioides*, enhanced its antioxidant enzyme activities, and decreased Cd and Pb concentrations in roots and shoots. Compared with CK, the addition of 4% MPAL, cultivated with *C. zizanioides*, reduced DTPA-extracted Cd and Pb in soils by 89.22% and 51.18%, respectively, at the highest level (p < 0.05). Moreover, urease, cellulase, and sucrase activities in soils treated with MPAL and cultivated with *C. zizanioides* were enhanced, with maximum increases of 37.70%, 110.22%, and 42.99%, respectively (p < 0.05). The interaction of MPAL and *C. zizanioides* increased bacterial richness and diversity but did not alter the bacterial community. This study demonstrated that the use of MPAL in combination with *C. zizanioides* could serve as a potential strategy for Cd and Pb immobilization and improvement of soil microecological properties.

## Graphical Abstract

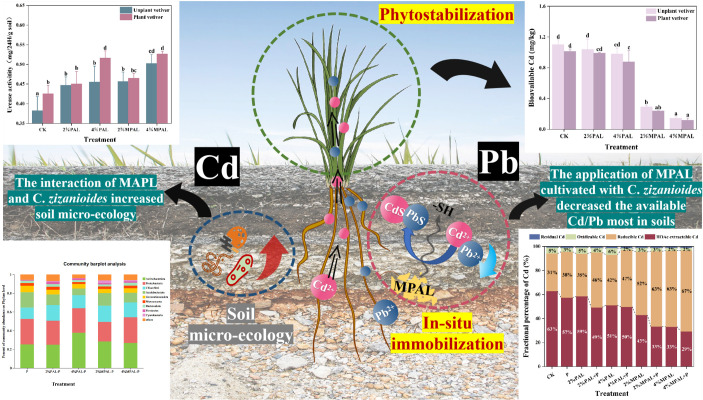

## 1 Introduction

Soil heavy metal pollution has become a worldwide environmental concern (Alam et al., 2024). Heavy metals in soils pose significant ecological risks, resulting in soil fertility decline and plant damage and ultimately transferring into the human body through the food chain (Azeez et al., 2019; Gravand et al., 2020; El-Sharkawy et al., 2023; Kama et al., 2024). Mining and smelting activities are among the major sources of heavy metal contamination, where metals frequently accumulate in soils at concentrations far exceeding regulatory thresholds (Cuypers et al., 2010; Antoniadis et al., 2019). Among these metals, cadmium (Cd) and lead (Pb) are particularly problematic due to their high toxicity and mobility, which have caused severe harm to both human health and the environment. While numerous studies have investigated remediation approaches for single-metal contamination (e.g., Cd or Pb), most mine-affected soils are simultaneously contaminated with Cd and Pb. Remediation strategies effective for individual metals often prove inadequate in multi-metal scenarios (Liu et al., 2000; Huang et al., 2009). More importantly, the immobilization mechanisms of Cd and Pb differ (Yang et al., 2022b), making it especially challenging to simultaneously stabilize both metals. Therefore, there is an urgent need to develop cost-effective and eco-friendly remediation strategies suitable for Cd and Pb co-contamination.

Phytostabilization has been recognized as a gentle remediation option that employs heavy metal-tolerant plants, often assisted by soil amendments, to reduce the mobility and bioavailability of toxic metals in soils. The main mechanisms include metal adsorption and precipitation in the rhizosphere, complexation with root exudates and cell walls, and sequestration into root vacuoles, which together limit their translocation to shoots. Through these processes, phytostabilization minimizes heavy metal entry into the food chain and groundwater, while improving soil quality and stability (Madrid et al., 2008; Shackira and Puthur, 2019; Zhan et al., 2020). It has been widely considered in soil remediation due to its economic, eco-friendly, and sustainable nature (Baker et al., 1994; Ali et al., 2013; Zheng et al., 2024). However, conventional heavy metal-tolerant plants are limited by biomass and growth conditions, resulting in low remediation efficiency (Shen et al., 2022). *Chrysopogon zizanioides* is a widespread perennial herb with a well-developed root system, high germination rates and substantial biomass production (George et al., 2017; Pandey and Praveen, 2020). In addition, *C. zizanioides* is highly adaptable to saline, calcareous, and nutrient-deficient soils, and can even tolerate high concentrations of heavy metals (Edelstein et al., 2009; Masinire et al., 2021). Recent studies have demonstrated that *C. zizanioides* has significant potential for Cd and Pb phytostabilization in soils (Ai et al., 2023). Pidatala et al. (2018) observed that the Pb uptake by *C. zizanioides* root reached 3.86 × 10^3^, 9.83 × 10^3^, 1.85 × 10^4^ mg·kg^-^¹ dry weight in Pb solutions at concentrations of 400 mg·L^-^¹, 800 mg·L^-^¹ and 1200 mg·L^-^¹, respectively. Previously, we also found that the concentration of Cd in *C. zizanioides* root was 18.4-fold higher than that found in the soil. In addition, it was reported that the ability of *C. zizanioides* to absorb Cd and Pb was greater under combined contamination conditions than under single-metal contamination (Ai et al., 2023).

Nevertheless, the biotoxicity of high concentrations of Cd and Pb in mining soils can hinder plant growth and reduce the phytostabilization efficiency of *C. zizanioides* in contaminated soils, and it has limited the application on mine rehabilitation. *In-situ* immobilization by applying active amendments to reduce the bioavailability of heavy metals has been proven to be an effective strategy to promote plant growth (Zheng et al., 2021; Lu et al., 2025). Common amendments, including biochar, liming, and phosphate mineral, reduce the bioavailability of Cd and Pb primarily by increasing soil pH (Song et al., 2022), but these amendments have little efficiency in alkaline soils (Yong et al., 2022). In this context, mercapto-modified palygorskite (MPAL) exhibits unique advantages in alkaline soils, where its surface mercapto groups can strongly bind Cd and Pb, effectively reducing metal bioavailability while maintaining soil chemical stability (Yong et al., 2024). Yang et al. (2022a) found that MPAL could significantly reduce the bioavailability of Cd in alkaline soils. The mercapto groups in the surface of MPAL have a strong affinity for Cd (*Ksp* of CdS = 8 × 10^-27^) and Pb (*Ksp* of PbS = 3.4 × 10^-28^). Our previous studies have demonstrated that MPAL application enhanced the microecology of Cd contaminated soils by improving the diversified sulfur metabolism biomarkers and decreasing the Cd resistance genes (Li et al., 2022). In addition to reducing Cd and Pb bioavailability, MPAL application could increase the abundance of plant growth-promoting bacteria thereby promoting plant growth. The evidence indicated that MPAL-assisted phytostabilization could provide an innovative approach to achieve Cd and Pb immobilization within a short time, especially in alkaline soils.

Herein, we hypothesize that MPAL application could provide a novel approach that simultaneously promotes C. zizanioides growth, immobilizes Cd and Pb, and improves rhizospheric microecology, thereby facilitating Cd and Pb phytostabilization in contaminated alkaline soils. The primary objectives of this study were to evaluate the effects of varying MPAL concentrations on Cd and Pb phytostabilization by analyzing plant biomass, antioxidant enzyme activities, Cd and Pb contents in plants, and soil Cd and Pb bioavailability and species distribution. Meanwhile, the effects of MPAL and C. zizanioides on rhizospheric microecology, including soil pH, enzyme activities, and microbial diversity and composition, were also examined.

## 2 Materials and methods

### 2.1 Soil and materials

The soil used in the experiment was collected from farmland in Chengdu, Sichuan Province, China. The collected soil samples were air-dried then sieved through a 2 mm mesh. After that, CdCl_2_ and Pb(NO_3_)_2_ solution were added, and the final Cd and Pb concentrations were 3 mg·kg^-^¹ and 700 mg·kg^-^¹, respectively, which represent typical levels of Cd and Pb contamination in mining soils and have been reported in previous studies (Zhou et al., 2024; Setu and Strezov, 2025). The spiked soils were incubated for a period to allow the metals to equilibrate with the soil matrix. This “aging” process ensured that the bioavailability and environmental behavior of Cd and Pb were similar to those in naturally contaminated soils, providing a reliable basis for subsequent remediation experiments. Pre-analytical assessments were conducted to confirm that the target concentrations of Cd and Pb had been successfully achieved. The resulting artificially contaminated soil was designated as the CK treatment (containing added Cd and Pb, without any amendments or plants) and served as the baseline control for all subsequent pot experiments.

The natural PAL was purchased from the Lingshou Qiangdong Mineral Processing factory (Shijiazhuang, China). MPAL was synthesized according to the method described by Fu et al. (2021). The successful mercapto functionalization of palygorskite (MPAL) was confirmed in our previous study (Li et al., 2022) by EDS analysis, which revealed distinct sulfur peaks with uniform distribution on the MPAL surface. FTIR and XRD analyses further verified the incorporation of mercapto groups while preserving the mineral framework. The Academy of agricultural science of Jiangsu province offered *C. zizanioides* seeds. All reagents used in this study were of analytical grade.

### 2.2 Pot experiment design

The pot experiment was conducted in the Chengdu University of Technology greenhouse. Ten different treatments were designed as follows: CK (Contaminated soil without treatment), 2% PAL (Contaminated soil treated with 2% PAL), 4% PAL (Contaminated soil treated with 4% PAL), 2% MPAL (Contaminated soil treated with 2% MPAL), 4% MPAL (Contaminated soil treated with 4% MPAL), P (Contaminated soil treated with plant), 2% PAL + P (Contaminated soil treated with 2% PAL and plant), 4% PAL + P(Contaminated soil treated with 4% PAL and plant), 2% MPAL + P (Contaminated soil treated with 2% MPAL and plant), 4% MPAL + P (Contaminated soil treated with 4% MPAL and plant). Each treatment was performed with three biological replicates, that is, three independent pots per treatment. Each pot was filled with 3 kg of aged soil, and the soil was thoroughly mixed with PAL and MPAL in different mass percentages according to treatment conditions. Seeds of *C. zizanioides* were germinated in a constant temperature incubator at 25°C, and 20 of healthy and uniform sprouts were transplanted into each pot. *C. zizanioides* were grown in a greenhouse at a temperature range of 25–28°C, watered daily with deionized (DI) water to 60% of the field water capacity, and harvested after 60 days.

### 2.3 Plant analysis

#### 2.3.1 Plant growth response

The *C. zizanioides* were carefully harvested and rinsed with DI water. The fresh and dry biomass were weighed. Then, 0.1 g of leaves were ground in 2 mL of phosphate buffer solution (PBS, pH=7.8) with liquid nitrogen, and then the homogenate was centrifuged at 4°C (10000 rpm, 10 min) to obtain enzyme solution. After that, peroxidase (POD) activity was determined using guaiacol as a substrate (Liao, 2015). Superoxide dismutase (SOD) activity was determined using nitroblue tetrazolium (NBT) (Durak et al., 1993). Catalase (CAT) activity was determined by monitoring the decrease in absorbance at 240 nm caused by H_2_O_2_ consumption (Kolahi et al., 2020). Soluble protein content was measured using the Coomassie brilliant blue method (Bradford, 1976). The hydrogen peroxide content was measured following the method of Uchida (Uchida et al., 2002). The production rate of superoxide anion radical was measured using the hydroxylamine hydrochloride oxidation method.

#### 2.3.2 Heavy metal analysis

0.1 g of dried plant powder was weighed and digested in a mixture of HNO_3_, HF, and HClO_4_ (5:5:3, v/v/v) at 180°C using an electric hot plate. Then, the digested solution was diluted to a final volume of 10 mL with 1% HNO_3_ (Wu et al., 2018). The mixture was centrifuged at 3000 rpm for 20 min, and the Cd and Pb concentrations in supernatants were measured by atomic absorption spectroscopy (AAS). Method blanks, duplicate samples, and certified reference materials (CRMs) were included to ensure reliability. Recoveries of CRMs ranged from 90% to 105%, and relative standard deviations (RSDs) for replicates were generally below 15%.

### 2.4 Soil analysis

#### 2.4.1 Soil pH and heavy metals

Soil pH was measured using a pH meter (METTLER-S220). The available heavy metal was extracted by using diethylenetriamine pentaacetic acid (DTPA). Metal fractions were extracted using the BCR sequential extraction procedure to evaluate metal bioavailability and transformation (Wu et al., 2018). Briefly: 1) 1 g of air-dried soil was mixed with 40 mL of 0.1 M CH_3_COOH and shaken at 150 rpm at 25 °C for 16 hours. After centrifugation, the supernatant was collected as the HOAc-extractable fraction, and the remaining soil was retained for the next step. 2) The retained soil was mixed with 40 mL of 0.5 M NH_4_OH·HCl (pH=2.0) and shaken at 150 rpm, 25°C for 16 h. After centrifugation, the supernatant was collected as the reducible fraction, and the residue was retained for the next step. 3) The residue was heated with 10 mL of 30% H_2_O_2_ (pH 2–3, 85°C) in a water bath until the solution volume was reduced to less than 1 mL. Then, 50 mL of 1 M NH_4_AC (pH=2) was added and shaken at 150 rpm, 25°C for 16 hours. After centrifugation, the supernatant was collected as the oxidizable fraction, and the residue was retained for the final step. 4) Finally, the remaining residue was digested in the same way as plant samples to obtain the residual fraction.

#### 2.4.2 Soil enzyme activity analysis

In this study, the activities of urease, catalase, sucrase and cellulase were determined. The activity of urease was determined by sodium phenol-sodium hypochlorite colorimetric method, the activity of catalase was measured by potassium permanganate titration, the activities of sucrase and cellulase were determined by the chromogenic reaction of 3, 5-dinitrosalicylic acid at 540 nm (Jiang et al., 2022).

#### 2.4.3 Microbial diversity analysis

The DNA from the soil sample was extracted with soil DNA extraction kit (Omega, Norcross, GA, U.S.), PCR amplification of 16S rRNA gene was performed with using universal primer pairs (343F: 5′-TACGGRAGGCAGCAG-3**’**; 798R: 5′-AGGGTATCTAATCCT-3′). QIIME2 was employed to analyze microbial sequencing data. The data of microbial community composition and alpha diversity were processed and analyzed through in the MajorBio cloud platform and then visualized by Origin 2021 software. Functional genes associated with sulfur metabolism was predicted from 16S rRNA gene data using the PICRUSt2 program, with functional annotations derived from the KEGG databases (**Supplementary Figure S1**).

### 2.5 Data analysis

In the experiment, each treatment was established with three biological replicates. The bioaccumulation factor (BCF) and translocation factor (TF) were calculated based on the methods of Yong et al. (2022) using Equations 1–3.


(1) $$\text{BCF}=\frac{heavy\;metal\text{ concentration }in\text{ plant}}{heavy\;metal\text{ concentration }in\;soil}$$

(2) $${\text{TF}}_{1}=\frac{heavy\;metal\;concentration\;in\;stem}{heavy\;metal\;concentration\;in\;root}$$

(3) $${\text{TF}}_{2}=\frac{heavy\;metal\;concentration\;in\;leaf}{heavy\;metal\;concentration\;in\;stem}$$

The mean and standard deviation were calculated using IBM SPSS Statistics 25, statistical significance was assessed using one-way ANOVA and Duncan’s multiple range test, with a significance level of 5%.

## 3 Results and discussion

### 3.1 Effect on soil pH

**Figure 1:**
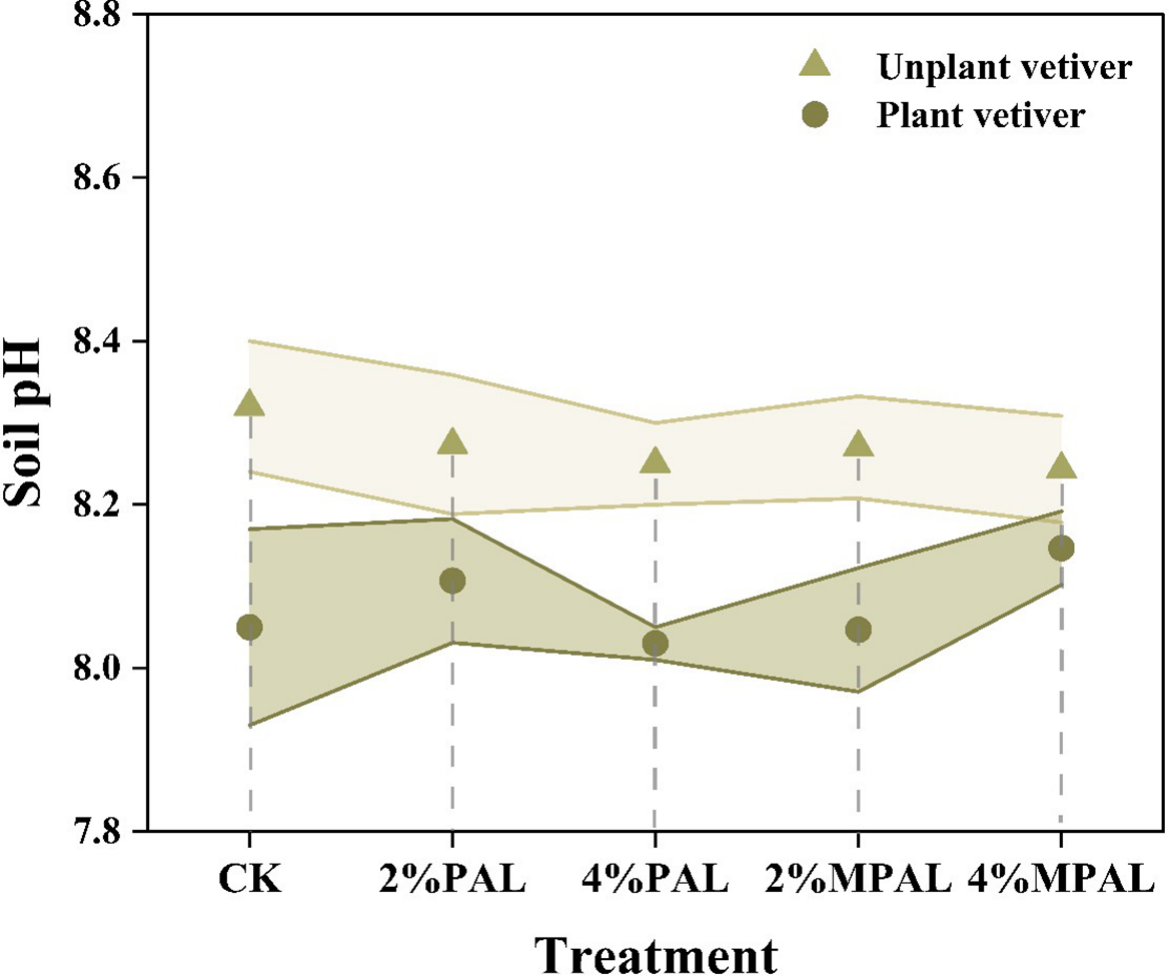
Soil pH under different treatments.

Soil pH is a crucial factor affecting the bio-availability of heavy metals. Most amendments, such as lime, biochar, and manure have been shown to enhance soil pH to reduce the bioavailable heavy metals (Ruttens et al., 2010; Wang et al., 2014). However, the significant variation in pH can markedly affect the soil micro-ecology, including soil enzyme activities, microbial communities and functions (Jiang et al., 2021; Naz et al., 2022). **Figure 1** illustrated that the application of PAL and MPAL has no significant effect on soil pH. This suggested that MPAL met the criteria for being environmentally friendly without changing soil pH. However, the cultivation of *C. zizanioides* resulted in a decrease in soil pH by 0.10 to 0.27. In general, plants can secrete low molecular weight acid (LMWA) to mobilize insoluble minerals to acquire essential nutrients, resulting in soil acidification (Marschner et al., 1987). A reduction in soil pH can increase the bioavailability of heavy metals, thus promoting the accumulation of heavy metals by plants (Zeng et al., 2011).

### 3.2 Effect on soil Cd and Pb availability and geochemical fractions

**Figure 2:**
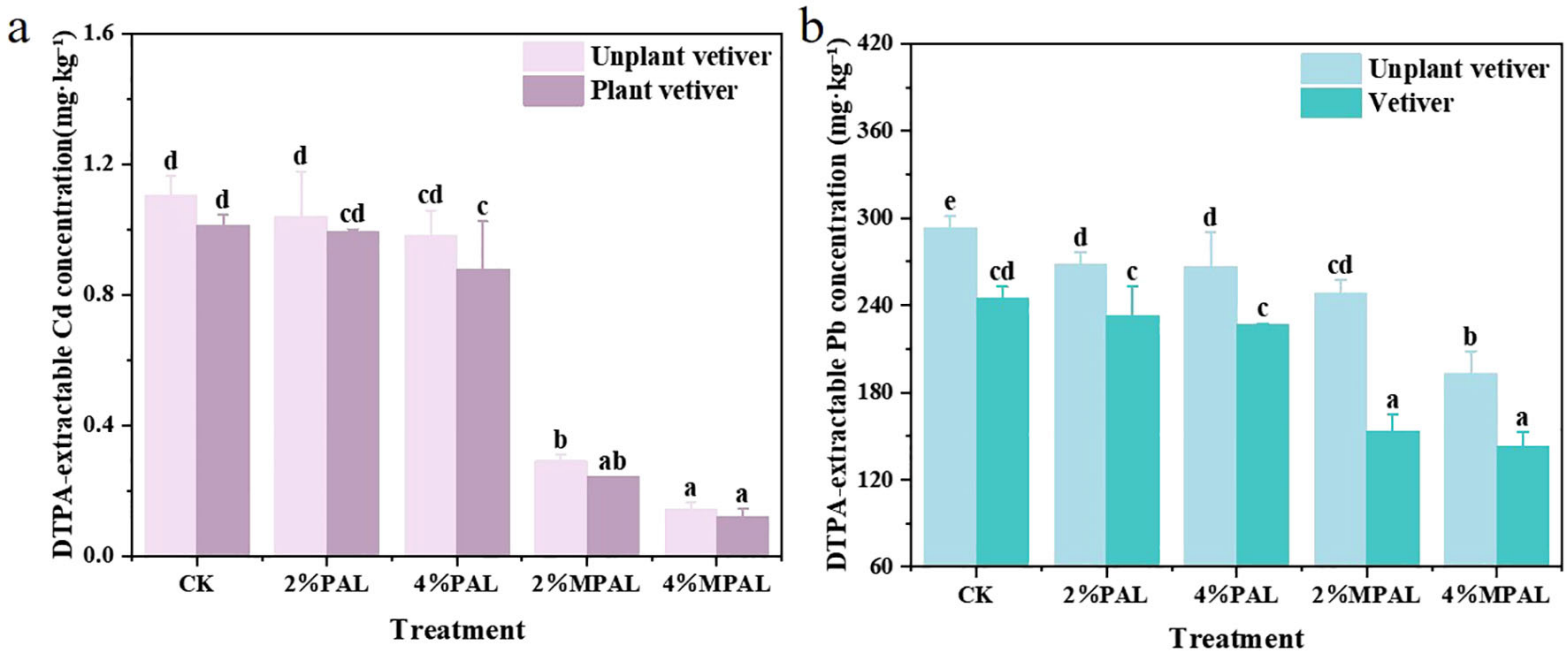
DTPA-extractable (bioavailable) Cd **(a)** and Pb **(b)** concentrations in soils under different treatments. Mean with different letters indicated significant difference from each other according to one-way ANOVA and Duncan’s test (p < 0.05).

As shown in **Figure 2A**, in the absence of *C. zizanioides* planting, the content of DTPA-extracted Cd in CK was 1.10 mg·kg^-^¹, which decreased by 5.73% - 10.96% and 73.54% - 86.93% in the PAL and MPAL treatments, respectively. Following the planting of *C. zizanioides*, the DTPA-extracted Cd further decreased by 4.63% - 17.48%. Most notably, the DTPA-extracted Cd showed the greatest decrease in the treatment of 4% MPAL with *C. zizanioides* planting, which was 89.22% lower than CK. As shown in **Figure 2B**, the content of DTPA-extracted Pb in CK was 293.26 mg·kg^-^¹, and the application of PAL and MPAL resulted in a significant reduction in the DTPA-extracted Pb content by 8.46% - 9.01% and 15.45% - 34.17%, respectively. The planting of *C. zizanioides* further decreased DTPA-extracted Pb by 13.31% - 38.18%.

**Figure 3:**
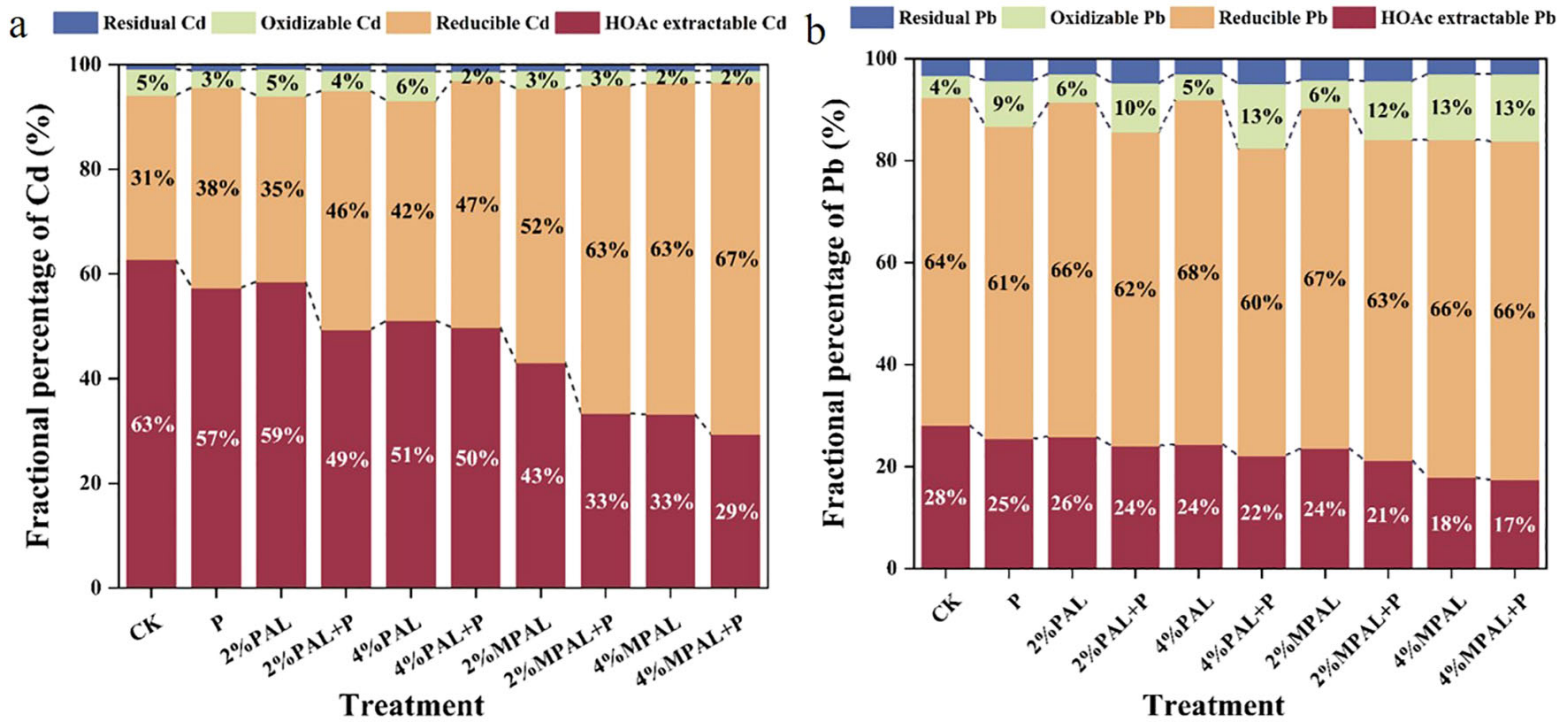
Speciation analysis of Cd **(a)** and Pb **(b)** under different treatments.

The various geochemical fractions of Cd and Pb were measured, and the results are presented in **Figure 3**. The application of MPAL and *C. zizanioides* decreased the proportion of HOAc-extractable Cd and increased the proportion of reducible Cd. Under the treatment of 4% MPAL and *C. zizanioides*, the HOAc-extractable Cd content decreased significantly by 53.71%, while the reducible Cd content increased markedly by 111.55%. Similarly, MPAL-assisted *C. zizanioides* incubation decreased the proportion of HOAc-extractable Pb and increased the proportions of reducible Pb and oxidizable Pb. Under the treatment of 4% MPAL and *C. zizanioides*, the proportion of HOAc-extractable Pb decreased significantly by 37.58%, while the proportion of oxidizable Pb increased markedly by 207.43%. These results showed that the application of MPAL assisted *C. zizanioides* can effectively immobilize Cd and Pb in the co-contaminated soil.

The potential immobilization mechanisms might be explained through the following aspects. Firstly, previous studies have shown that MPAL can effectively immobilize Cd and Pb by adsorption, ion - exchange and co-precipitation (Wu et al., 2023). The surface of MPAL contains plentiful functional groups, including -OH, -COOH, and -CH, which can provide adsorption sites for Cd and Pb (Kumpiene et al., 2008). The sulfhydryl groups of MPAL can target bind Cd and Pb to form stable CdS and PbS complexes. Secondly, *C. zizanioides* can immobilize abundant Cd and Pb irons by its developed root system, thus decreasing the bioavailability of Cd and Pb in soils (Rotkittikhun et al., 2007; Banerjee et al., 2016). Thirdly, the application of MPAL reduced the metal toxicity, and the enhancement of plant biomass further promoted the reduction of bioavailable Cd and Pb (Li et al., 2022).

### 3.3 Effect on physiological characteristics of *C. zizanioides*

The effects of soil amendments on the biomass of *C. zizanioides* grown in Cd and Pb contaminated soils are shown in **Table 1**. Compared to CK, the application of PAL and MPAL has positive effects on plant growth. Especially, in the 4% MPAL treatment, the dry weight of root, stem and leaf observably increased by 35.71%, 71.43% and 82.46%, respectively. These results indicated that MPAL could significantly promote *C. zizanioides* growth in the Cd and Pb contaminated soil.

**Table 1:** The biomass of *C. zizanioides* under the PAL and MPAL treatments.

Treatments	Fresh weight			Dry weight		
	Roots (g)	Stems (g)	Leaves (g)	Roots (g)	Stems (g)	Leaves (g)
CK	0.20 ± 0.14 a	0.61 ± 0.21 a	2.31 ± 0.05 a	0.14 ± 0.07 a	0.14 ± 0.05 a	0.57 ± 0.13 a
2% PAL	0.21 ± 0.07 a	0.60 ± 0.22 a	2.66 ± 0.99 ab	0.16 ± 0.02 a	0.15 ± 0.04 a	0.72 ± 0.15 ab
4% PAL	0.27 ± 0.06 a	0.63 ± 0.22 a	3.37 ± 0.81 ab	0.18 ± 0.04 a	0.17 ± 0.02 a	0.83 ± 0.32 ab
2% MPAL	0.23 ± 0.01 a	0.64 ± 0.07 a	3.23 ± 0.38 ab	0.16 ± 0.00 a	0.16 ± 0.01 a	0.72 ± 0.11 ab
4% MPAL	0.31 ± 0.03 a	0.73 ± 0.07 a	3.57 ± 0.10 b	0.19 ± 0.02 a	0.24 ± 0.00 b	1.04 ± 0.20 b

Values within a column followed by different lowercase letters are significantly different at p < 0.05 according to Duncan’s test.

Heavy metals can induce plants to produce superoxide anion radicals (O_2_·-) and hydrogen peroxide (H_2_O_2_), which are important components of reactive oxygen species (ROS) in plants. Excessive O_2_·- and H_2_O_2_ can lead to oxidative damage to plant tissues. In response to oxidative stress caused by heavy metals, plants produce various types of antioxidants to eliminate ROS, such as SOD, POD and CAT (Ali et al., 2013; Ben et al., 2023). Therefore, the antioxidant enzyme activities are critical protective mechanisms under heavy metal stress in plants, and the degree of oxidative stress in plants can be assessed by measuring the antioxidant enzyme activities (Luna et al., 1994; Ahmad et al., 2011; Zulfiqar et al., 2019). In this study, the production rate of O_2_-·, the H_2_O_2_ content, and soluble protein content increased under PAL and MPAL treatments (except for 2% PAL) (**Table 2**). In the 4% PAL treatment, the production rate of O_2_·- was twice that of the CK group. The highest soluble protein contents and H_2_O_2_ content were observed in the 2% MPAL treatment, which were 15.88% and 633.33% higher than those in the CK group. Correspondingly, the application of MPAL significantly enhanced the SOD and POD activities by 8.55% - 9.12% and 29.85% - 70.14%, respectively, compared to the CK group. In addition, the activity of CAT in the 4% PAL and 4% MPAL was increased by 16.40% and 22.61%, respectively. This phenomenon showed that the addition of MPAL enhanced the antioxidant capacity of *C. zizanioides* under the stress of heavy metals.

**Table 2:** The physiological characteristics of *C. zizanioides* under the PAL and MPAL treatments.

Treatments	SOD activity (U·g^-^¹·FW)	POD activity (U·g^-^¹·min^-^¹·FW)	CAT activity (U·g^-^¹·min^-^¹·FW)	Soluble protein (mg·g^-^¹·FW)	Production rate of O_2_·- (nmol·g^-^¹·min^-^¹·FW)	H_2_O2 (mmol·g^-^¹·FW)
CK	2138.84 ± 40.45 a	63.81 ± 6.67 a	14510 ± 632 a	25.69 ± 2.10 ab	0.11 ± 0.02 a	1467 ± 652 a
2% PAL	2122.65 ± 4.86 a	57.14 ± 5.71 a	16620 ± 1242 b	21.86 ± 2.64 a	0.12 ± 0.01 a	2119 ± 978 a
4% PAL	2051.41 ± 95.22 a	62.86 ± 0.00 a	16890 ± 1301 b	27.76 ± 2.36 b	0.22 ± 0.03 c	6237 ± 432 b
2% MPAL	2321.80 ± 31.85 b	108.57 ± 3.81 c	14685 ± 75 a	29.77 ± 1.50 b	0.21 ± 0.02 c	10758 ± 489 c
4% MPAL	2333.94 ± 104.43 b	82.86 ± 2.86 b	17790 ± 1470 b	26.75 ± 2.24 b	0.16 ± 0.02 b	6765 ± 408 b

Values within a column followed by different lowercase letters are significantly different at p < 0.05 according to Duncan’s test.

### 3.4 Effect on the uptake of Cd and Pb by *C. zizanioides*

The uptake and accumulation of Cd and Pb by the roots, stems, and leaves of *C. zizanioides* are shown in **Table 3**. Compared to CK, the application of PAL had no significant effect on the uptake of Cd and Pb by *C. zizanioides*. However, the application of MPAL observably reduced the uptake of Cd and Pb by *C. zizanioides*. The Cd content of roots, stems and leaves under the MPAL treatment mostly decreased by 95.06%, 54.54% and 37.55%, respectively, compared to CK group. Meanwhile, the Pb content of roots, stems and leaves decreased by 31.71% - 63.58%, 16.48% - 24.03%, and 22.35% - 23.87%, respectively.

**Table 3:** Heavy metal concentrations (mg·kg^-^¹) in plant tissues under the PAL and MPAL treatments.

Treatments	Cd			Pb		
	Roots(mg·kg^-^¹)	Stems(mg·kg^-^¹)	Leaves(mg·kg^-^¹)	Roots(mg·kg^-^¹)	Stems(mg·kg^-^¹)	Leaves(mg·kg^-^¹)
CK	32.41 ± 5.59 b	11.02 ± 0.07 c	5.22 ± 0.19 b	1164.05± 0.00 c	59.63 ± 4.10 ab	76.69 ± 10.20 c
2% PAL	45.97 ± 3.38 c	10.92 ± 1.32 c	6.73 ± 0.15 c	1052.55± 136.35 c	66.34 ± 12.48 b	71.73 ± 5.48 ab
4% PAL	31.61 ± 1.47 b	9.16 ± 0.87 b	3.08 ± 0.12 a	886.69± 90.26 b	62.14 ± 13.87 ab	71.60 ± 7.57 ab
2% MPAL	1.60 ± 0.07 a	5.13 ± 0.33 a	5.65 ± 0.91 b	794.95± 35.95 b	49.80 ± 2.21 ab	58.38 ± 6.19 a
4% MPAL	1.72 ± 0.14 a	5.01 ± 0.11 a	3.26 ± 0.14 a	423.95± 2.56 a	45.30 ± 8.92 a	59.55 ± 4.26 a

Values within a column followed by different lowercase letters are significantly different at p < 0.05 according to Duncan’s test.

We observed abnormally high concentrations of Cd (32.41 mg·kg^-^¹) and Pb (1164.05 mg·kg^-^¹) in the roots of the CK treatment. This phenomenon can be attributed to the relatively high background levels of Cd (3 mg·kg^-^¹) and Pb (700 mg·kg^-^¹) in the soil, together with the strong enrichment capacity of *C. zizanioides*. Previous studies have similarly reported excessive root accumulation of heavy metals by this species. For instance, the study by Punamiya et al. (2010) showed that in Pb-contaminated soil (800 mg·kg^-^¹), Pb concentration in *C. zizanioides* roots reached nearly 2000 mg·kg^-^¹. Likewise, Zhang et al. (2014) found that at a soil Cd concentration of 15 mg·kg^-^¹, Cd accumulation in the roots of *C. zizanioides* reached as high as 167 mg·kg^-^¹. These results consistently demonstrate the remarkable root accumulation capacity of *C. zizanioides*, which often exceeds the nominal soil concentrations. In our study, this characteristic explains the unusually high Cd and Pb levels observed in CK roots, confirming that the root system plays a dominant role in metal sequestration and thereby contributes to phytostabilization.

Moreover, the BCF and TF values of Cd and Pb were calculated to reveal the migration of Cd and Pb in the soil-plant system (**Table 4**). It was observed that the BCF of Cd decreased significantly from 10.80 to 0.57 and 0.53 with the addition of 2.0% and 4.0% MPAL, respectively, and the BCF of Pb decreased from 1.66 to 0.70 with 4% MPAL addition. However, the addition of MPAL increased the TF values of Cd, but had no obvious effect on the TF values of Pb. The reduction of Cd and Pb in *C. zizanioides* can be explained by the decrease in bioavailable Cd and Pb in soil (**Figure 2**). The bioavailability of Cd and Pb in soil is a vital factor to affect the uptake and translation of Cd and Pb by plants (Li et al., 2019). The MPAL application reduced the DTPA-extracted Cd and Pb and enhanced the proportion of reducible and oxidizable Cd and Pb, thereby decreasing the Cd and Pb contents in *C. zizanioides*.

**Table 4:** The bioaccumulation factors (BCF) and translocation factors (TF) of *C. zizanioides* under the PAL and MPAL treatments.

Treatments	Cd			Pb		
	BCF	TF_1_	TF_2_	BCF	TF_1_	TF_2_
CK	10.80 ± 1.86 b	0.35 ± 0.05 a	0.47 ± 0.02 ab	1.66 ± 0.00 c	0.05 ± 0.00 a	1.28 ± 0.15 ab
2% PAL	15.32 ± 1.12 c	0.24 ± 0.03 a	0.62 ± 0.08 bc	1.50 ± 0.19 c	0.06 ± 0.01 a	0.93 ± 0.03 a
4% PAL	10.54 ± 0.49 b	0.29 ± 0.04 a	0.34 ± 0.04 a	1.27 ± 0.13 b	0.08 ± 0.00 b	1.18 ± 0.16 ab
2% MPAL	0.53 ± 0.02 a	3.22 ± 0.22 c	1.10 ± 0.18 d	1.14 ± 0.05 b	0.06 ± 0.00 a	1.17 ± 0.10 ab
4% MPAL	0.57 ± 0.05 a	2.93 ± 0.24 b	0.65 ± 0.04 c	0.70 ± 0.16 a	0.10 ± 0.02 c	1.37 ± 0.39 ab

Values within a column followed by different lowercase letters are significantly different at p < 0.05 according to Duncan’s test.

The observed increase in Cd TF under MPAL treatments might be attributed to two main mechanisms. First, although MPAL significantly reduced Cd content in roots, plants may regulate the activity of metal transport proteins, such as HMA2 and HMA4, to preferentially translocate the remaining Cd from roots to shoots, resulting in higher TF values. Second, MPAL may alter the speciation or binding forms of Cd in roots, making the residual Cd more readily available for xylem loading and long-distance transport to aboveground tissues (Raza et al., 2020). Together, these mechanisms explain why root Cd decreased markedly while shoot Cd was only moderately reduced, leading to an apparent increase in TF.

### 3.5 Effect on soil enzyme activities

**Figure 4:**
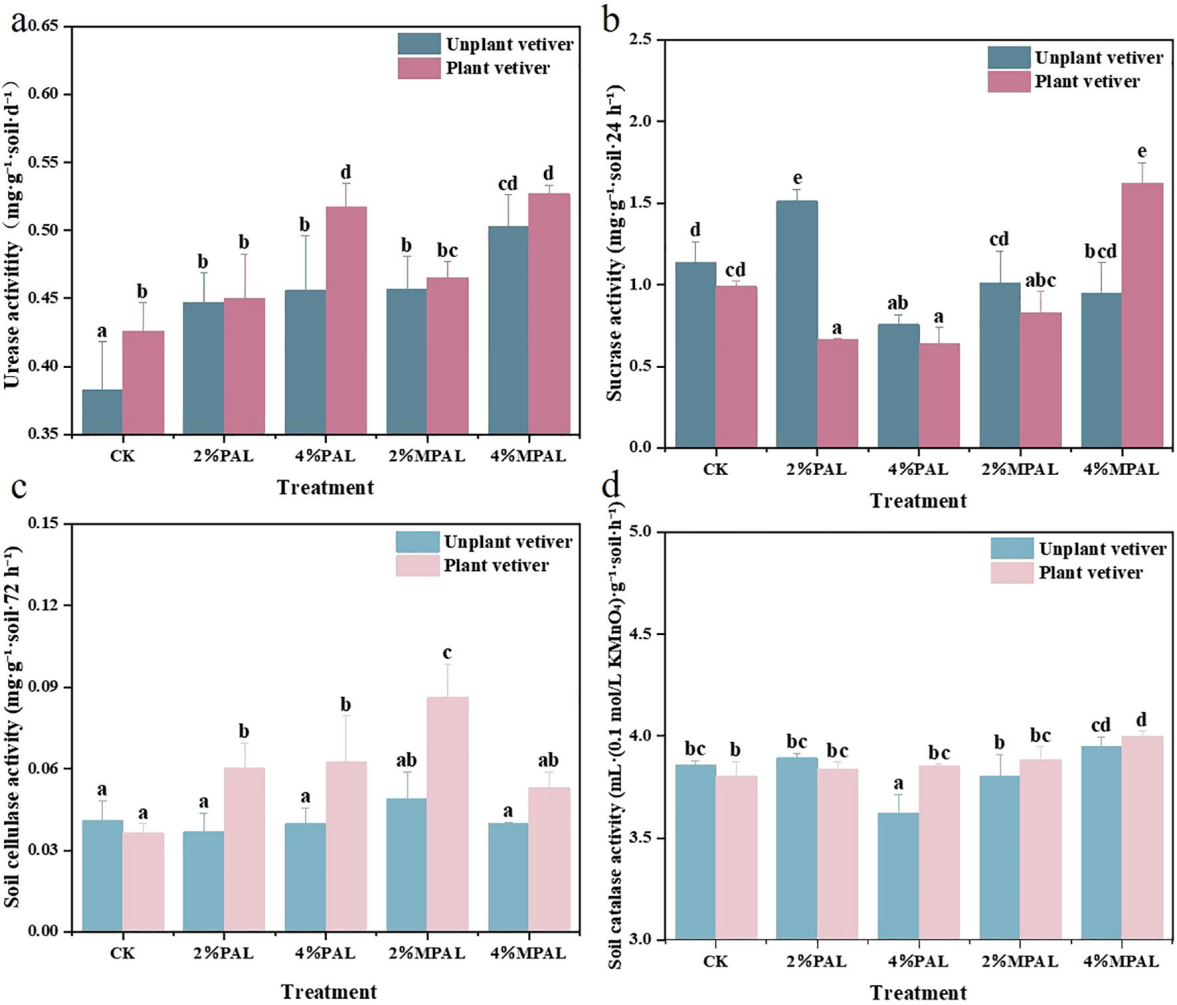
Soil urease activity **(a)**, sucrase activity **(b)**, cellulase activity **(c)** and catalase activity **(d)** under different treatments. Mean with different letters indicated significant difference from each other according to one-way ANOVA and Duncan’s test (p < 0.05).

Soil enzyme activities are widely used to reflect soil biochemical properties and assess remediation efficiency (Pu and Liu, 2023). In this study, urease, catalase, cellulase, and sucrase activities were determined to estimate the biological properties of soil after the remediation with amendments and *C. zizanioides* (**Figure 4**). It was observed that both amendments and *C. zizanioides* increased urease activity. The application of PAL and MPAL increased the urease activity by 16.71% - 19.06% and 19.32% - 31.33%, respectively, compared to CK group. The maximum urease activity was observed in the treatment of 4% MPAL with planting *C. zizanioides*, which was 37.70% higher than CK. Previous studies have also shown that sulfydryl modified materials application can enhance the soil urease activity. As illustrated by Zhu et al, thiourea-modified biochar significantly enhanced the urease activity (Zhu et al., 2022). However, the application of PAL and MPAL alone had no significant effects on the activities of catalase, cellulase and sucrase, but the combined application of amendments and *C. zizanioides* enhanced the catalase and cellulase activities. Under the treatment of PAL and MPAL, planting *C. zizanioides* significantly enhanced the cellulase activity by 29.03% - 110.22% compared to CK. In addition, the application of 4% MPAL and *C. zizanioides* significantly increased the sucrase activity by 42.99%. The soil enzyme was secreted from plants and soil microorganisms. The MPAL application reduced the soil Cd and Pb bioavailability, and promoted *C. zizanioides* growth. The rhizosphere activity of *C. zizanioides* might recruit more microorganisms and enhance the enzyme activities.

### 3.6 Effects on soil bacterial community

In this study, the effects of soil amendments and the plant *C. zizanioides* on bacterial community structure were analyzed using 16S rRNA gene sequencing. The richness and diversity of bacterial communities at the OTU level under different treatments are shown in **Supplementary Table S1**. The Ace and Chao indices reflect the richness of bacterial communities, while the Shannon and Simpson indices are used to evaluate their diversity (Wu et al., 2018). No significant variation in all indices was observed after the application of PAL and MPAL, indicating that their application has little negative impact on bacterial diversity. However, the *C. zizanioides* planting enhanced the bacterial Chao, Ace and Shannon indexes. The growth of *C. zizanioides* can recruit more beneficial microorganisms by root exudates.

**Figure 5:**
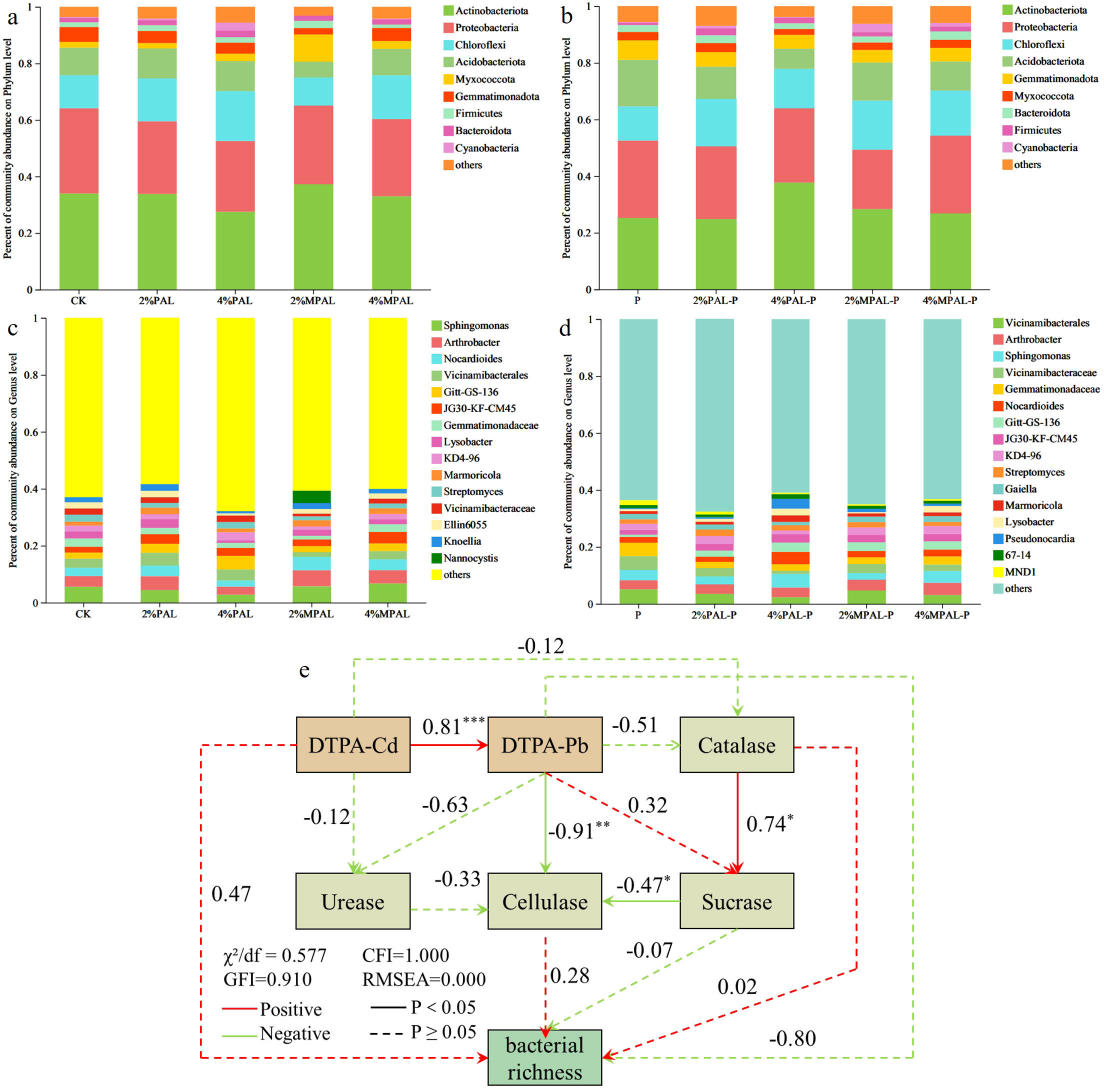
Community abundance on phylum level **(a, b)** and genus level **(c, d)** under different treatments. The structural equation model analysis between environmental factors and bacterial richness, numbers next to the arrows indicate standardized path coefficients and asterisks mark their significance: *<0.05; **<0.01; or ***<0.001 **(e)**.

**Figures 5a, b** shows that all treatments have a similar community composition at the phylum level. The dominant bacterial phyla were detected to be *Actinobacteriota*, *Proteobacteria*, *Chloroflexi* and *Acidobacteriota*, accounting for 78.62% - 85.56% of the total sequences. *Actinobacteriota* is an important phylum for metal remediation due to its function of metabolizing and promoting the absorption of heavy metals by plants (Alvarez et al., 2017; Narendrula-Kotha and Nkongolo, 2017). *Chloroflexi* can better adapt to heavy metal stress and participate in the metabolism of organic matter and the transformation of pollutants (Gupta et al., 2023). In the treatment of cultivation of *C. zizanioides*, the addition of PAL and MPAL increased the abundance of *Actinobacteriota* and *Chloroflexi*. Moreover, the abundance of *Actinobacteriota* and *Chloroflexi* is negatively correlated with the concentrations of heavy metals (Li et al., 2022; Wu et al., 2023), indicating that the incorporation of amendment reduces the toxicity of heavy metals and enhances soil microecology. *Acidobacteriota* was adapted to low pH condition (Qin et al., 2020), and it might be the reason why cultivation of *C. zizanioides* significantly increased the abundance of *Acidobacteriota* compared to the control. Other phyla were relatively stable in all treatments.

**Figures 5c, d** showed that the community composition at the genus level is significantly different. In the cultivation of *C. zizanioides* alone, *Vicinamibacterales*, *Vicinamibacteraceae* and *Gemmatimonadaceae* were dominant in the soil. As illustrated by Kou et al, *Vicinamibacterales* and *Vicinamibacteraceae* may be resistant to heavy metals, which may be the reason for this phenomenon (Kou et al., 2023). The addition of PAL and MPAL significantly decreased the abundance of *Vicinamibacteraceae* and *Gemmatimonadaceae*. We speculated that the application of amendments decreased the bioavailability of heavy metals in the soil could potentially account for this result. *Sphingomonas* is a gram-negative bacteria, which was sensitive to metal contamination stress (Wang et al., 2023). Without *C. zizanioides* planting, *Sphingomonas* was the dominant bacterial genus, after *C. zizanioides* planting, the abundance of *Sphingomonas* decreased slightly, indicating that the application of *C. zizanioides* reduces the bioavailability of heavy metals in soil.

The Structural Equation Model (SEM) was constructed to examine the causal relationships among DTPA-extractable Cd and Pb, soil enzyme activities (urease, catalase, cellulase), and bacterial richness (Sobs) (**Figure 5e**). The results indicated that heavy metals directly suppressed bacterial richness and indirectly influenced it through negative effects on soil enzyme activities. The SEM showed excellent fit (χ²/df = 0.58, CFI = 1.00, RMSEA = 0.00), confirming that the proposed pathways accurately reflect the observed relationships. Compared with simple correlation analysis, SEM provides a powerful tool that can distinguish the direct and indirect effects of multiple factors on contaminated soil (Li et al., 2025). Our research results indicate that the toxicity of Cd and Pb reduces the activity of soil enzymes, thereby restricting the turnover of organic matter and the cycling of nutrients, and ultimately inhibiting the diversity of microorganisms. Importantly, the MPAL treatment alleviates these negative effects by reducing the bioavailability of metals, demonstrating its direct and indirect benefits for the soil microecology. These findings emphasize that MPAL-assisted plant stabilization not only can fix metals but also can help maintain the functional connection between soil enzymes and microbial communities.

## 4 Conclusions

This study demonstrates that the application of mercapto-functionalized palygorskite (MPAL) effectively enhances the phytostabilization capacity of Chrysopogon zizanioides for Cd and Pb. The addition of MPAL significantly reduced the bioavailable fractions of Cd and Pb in the soil, with the 4% MPAL treatment cultivated with C. zizanioides decreasing DTPA-extractable Cd and Pb by 89.22% and 51.18%, respectively. Meanwhile, MPAL markedly promoted plant growth and improved soil biological properties, including enhanced soil enzyme activities and microbial richness. These findings indicate that MPAL-assisted phytostabilization is a promising strategy for the remediation of soils co-contaminated with Cd and Pb. However, this study was conducted under pot-scale conditions with artificially contaminated soils, which may not fully represent the complexity of field environments in mining areas. Future research should focus on field-scale validation to assess the long-term stability and remediation efficiency of MPAL-assisted phytostabilization, as well as its applicability under multi-metal co-contamination conditions.

## Data Availability

The original contributions presented in the study are publicly available. This data can be found at the National Center for Biotechnology Information (NCBI) using accession number PRJNA1284549.
