# Supplementary material for: Sustainable remediation of heavy metal contaminated soil through phytostabilization with the in-situ immobilization by mercapto-based palygorskite
Source: Front Plant Sci. 2025 Oct 24;16:1659418. doi: 10.3389/fpls.2025.1659418 (PMC12592181; doi:10.3389/fpls.2025.1659418)
Supplement: Supplementary file 1 [file DataSheet1.docx]

**Table S1** Microbial diversity indexs under different treatments.

**Figure S1.** Predicted functional gene abundance related to the sulfur metabolism under different treatments (from left to right: CK, 2% MPAL, P, 2% MPAL+P), based on 16S rRNA gene sequencing data. The heatmap indicates the relative abundance of key sulfur metabolism genes (red: high abundance, blue: low abundance).

**TableS1** Microbial diversity indexs under different treatments.

| Treatments | | Ace | Chao | Coverage | Shannon | Simpson | Sobs |
| --- | --- | --- | --- | --- | --- | --- | --- |
| Without *C. zizanioides* | CK | 3090.38 | 3074.80 | 0.98 | 6.31 | 0.0060 | 2572 |
|  | 2%PAL | 3008.78 | 2962.76 | 0.99 | 6.21 | 0.0074 | 2571 |
|  | 4%PAL | 3188.9 | 3225.15 | 0.98 | 6.41 | 0.0052 | 2629 |
|  | 2%MPAL | 3015.74 | 3056.84 | 0.99 | 5.85 | 0.0103 | 2453 |
|  | 4%MPAL | 31282.08 | 3094.08 | 0.98 | 6.30 | 0.0070 | 2715 |
| Plant *C. zizanioides* | CK | 2983.89 | 2968.08 | 0.99 | 6.51 | 0.0040 | 2534 |
|  | 2%PAL | 3433.34 | 3432.63 | 0.99 | 6.63 | 0.0039 | 3004 |
|  | 4%PAL | 3052.35 | 3109.21 | 0.99 | 6.21 | 0.0061 | 2490 |
|  | 2%MPAL | 3468.63 | 3426.59 | 0.99 | 6.58 | 0.0043 | 2992 |
|  | 4%MPAL | 3321.46 | 3316.96 | 0.99 | 6.44 | 0.0053 | 2780 |


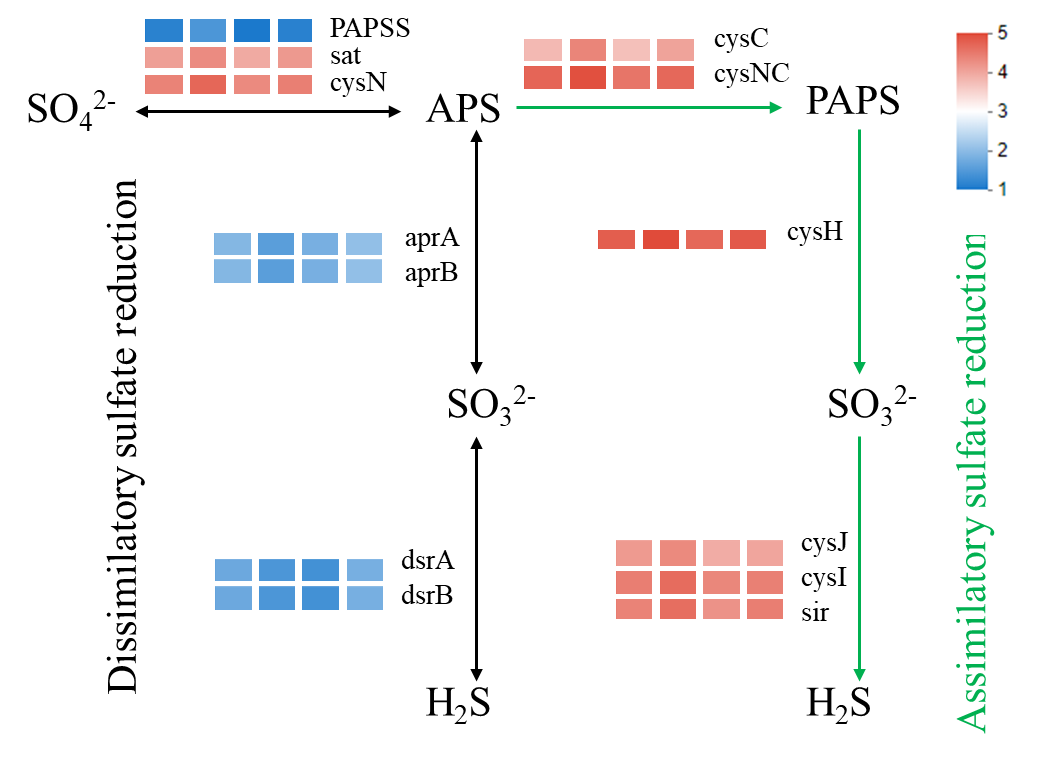


**Figure S1.** Predicted functional gene abundance related to the sulfur metabolism under different treatments (from left to right: CK, 2% MPAL, P, 2% MPAL+P), based on 16S rRNA gene sequencing data. The heatmap indicates the relative abundance of key sulfur metabolism genes (red: high abundance, blue: low abundance).
